# Factors Affecting Continuous Renal Replacement Therapy (CRRT) in Patients With Septic Shock: An Analysis of a National Inpatient Sample Database

**DOI:** 10.7759/cureus.74356

**Published:** 2024-11-24

**Authors:** Karan Yagnik, Gaurav Mohan, Apurva Ketkar, Noel Nivera, Sharon Weiner, Chandler Patton, Doantrang Du

**Affiliations:** 1 Internal Medicine, Monmouth Medical Center, Long Branch, USA; 2 Nephrology, Monmouth Medical Center, Long Branch, USA; 3 Pulmonology, Rutgers Health/Monmouth Medical Center, Long Branch, USA; 4 Pulmonary and Critical Care, Monmouth Medical Center, Long Branch, USA; 5 Internal Medicine, Robert Wood Johnson (RWJ) Barnabas Health, Long Branch, USA

**Keywords:** aki outcome, continuous renal replacement therapy (crrt), critical care nephrology, icu patients, severe sepsis, surviving sepsis guidelines

## Abstract

Background: Septic shock is defined as sepsis with hypotension requiring vasopressors to maintain a mean arterial pressure above 65 mmHg and having a serum lactate level of more than 2 mmol/L despite adequate volume resuscitation as per the Sepsis-3 criteria. Continuous renal replacement therapy (CRRT) is commonly utilized in septic shock patients for the treatment of acute kidney injury as well as for modulating immune response and maintaining hemodynamic stability.

Methods: We looked at the National Inpatient Sample database in 2019. We identified adult patients with septic shock as the primary diagnosis using the International Classification of Diseases, 10th revision, clinical modification codes R65.21 and R78.81, and subbranches of Aa41, A40, and R60. STATA 18 (StataCorp, College Station, TX) was used to perform logistic multivariate regression analyses.

Results: A total of 15,794 adults who were admitted for septic shock as the primary diagnosis underwent CRRT. The mean age of the patients was 61.7 years. The overall mortality rate was 57% (N = 9,002). An increase in age by one year was associated with a 1% increase in mortality (p = 0.001). The presence of hypertension increased mortality by 29% (N = 6,391) (p = 0.028). Interestingly, preexisting diabetes mellitus improved mortality by 37% (N = 3331) (p = 0.001).The outcome of CRRT was better in patients with chronic kidney disease, with a 26% improvement in mortality (N = 2341) (p = 0.001). A significant improvement in outcome (29% decrease in mortality, p=0.013) and 31% reduction in hospital length of stay (p = 0.008) was noted with CRRT initiated on day 2 of hospitalization.

Conclusion: This study highlights that the approximate time of initiation of CRRT for optimal benefit of the treatment is between 24 and 48 hours of hospitalization. This study emphasizes the prognostic factors of a standard therapy, which can serve as a basis for clinical decision-making.

## Introduction

Septic shock is defined as sepsis with hypotension requiring vasopressors to maintain mean arterial pressure (MAP) above 65 mmHg and a serum lactate level of more than 2 mmol/L despite adequate volume resuscitation as per the Sepsis-3 criteria [1]. It has a hospital mortality rate of more than 40%. Renal injury occurs early in sepsis, and acute kidney injury (AKI) develops in about two-thirds of patients with septic shock [2].

Sepsis-associated acute kidney injury (S-AKI) is a common and life-threatening complication that increases hospital mortality and the risk of developing chronic kidney disease (CKD) by six to eight times and three times, respectively [3-5]. Continuous renal replacement therapy (CRRT) is commonly performed in S-AKI patients as, apart from conventional renal replacement functioning, it also utilizes immune modulation and maintains hemodynamic stability in septic shock patients, thus improving survival rate. These functions are more effective in CRRT than in intermittent hemodialysis [6,7].

Previous studies have indicated that male gender is linked to higher mortality rates [8]. Additionally, advanced age and hypertension (HTN) are recognized predictors of mortality. No actual guidelines are available regarding when to initiate CRRT, but previous records suggested early initiation (within one to two days) is associated with better outcomes than later initiation (greater than two days) [9,10]. However, considering other complications of CRRT, to better understand the outcomes of CRRT in patients with septic shock, we conducted a retrospective observational study using the national inpatient database containing all United States hospital admissions.

## Materials and methods

Study design and data source

This is a retrospective observational cohort study involving adult patients (age >18 years) who were hospitalized with the primary diagnosis of septic shock in United States hospitals between January 1, 2019, and December 31, 2019. Data were obtained from the National Inpatient Sample (NIS) database in 2019. The NIS is the largest publicly available inpatient database, covering more than 98% of the United States population and approximately 1,000 hospitals. It includes patient demographics, discharge status, all listed diagnoses (per the International Classification of Diseases 10th revision, ICD-10, clinical modification/procedure coding system), procedures, and inpatient charges.

Study population

The study included adult patients hospitalized with septic shock as the primary diagnosis using ICD-10 codes R65.21 and R78.81, subbranches of A41, A40, and R60, and undergoing CRRT. Patients were excluded if they were under 18 years of age, did not have septic shock, or did not undergo CRRT in the course of hospitalization. Subgroup analysis was done on patients who underwent CRRT on days 1-3 of hospitalization to compare the mortality rates. Patient characteristics and comorbidities were recorded in all groups.

Outcome measures

The primary outcome was comparing the mortality rate among the patients with septic shock undergoing CRRT. The secondary outcome was reducing the length of hospital stay as a measure of healthcare utilization cost.

Statistical analysis

STATA 18 (by StataCorp, College Station, TX) was used for data analysis. Logistic multivariate regression analyses were performed to adjust for possible confounders. Odds ratios were calculated to compare variations in outcome associated with patient demographic characteristics, comorbidities, and time to initiation of renal replacement therapy (RRT) since hospitalization. A p value of 0.05 was set as the threshold for statistical significance.

Ethical considerations

The NIS database does not contain any patient, hospital, or state-level identifiers. This is in accordance with Health Insurance Portability and Accountability Act regulations and respects patient protection and anonymity. Thus, all NIS-based studies are exempt from institutional review board approval.

## Results

A total of 15,794 adults who were admitted for septic shock as the primary diagnosis underwent CRRT. The mean age of the patients was 61.7 years. The overall mortality rate was 57% (N = 9,002). An increase in age by one year was associated with a 1% increase in mortality (p = 0.001). Gender, ethnicity, and congestive heart failure (CHF) did not have any significant association with the outcome. The presence of HTN increased mortality by 29% (N = 6,391) (p = 0.028). Interestingly, preexisting diabetes mellitus (DM) improved mortality by 37% (N = 3331) (p = 0.001). This finding could be supported by evidence that suggests that DM is associated with a lower risk of acute lung failure in septic shock and a higher risk of AKI, which, in turn, is directly managed with CRRT. The outcome of CRRT showed a 26% improvement in mortality among patients with CKD (N = 2341) (p = 0.001). However, the literature does not provide a satisfactory explanation for this observation. It could be attributed to multiple confounding variables, such as the severity of hypotension, sepsis-induced respiratory failure, and coexisting DM in CKD patients (Table 1).

**Table 1:** Mortality predictors (adjusted for confounders)

Risk factor	Hazard ratio	p value
Male gender	1.41	>0.05
Older age	1.01	0.001
African-American ethnicity	1.05	>0.05
Congestive heart failure	1.35	>0.05
Hypertension	1.29	0.028
Diabetes mellitus	0.63	0.001
Chronic kidney disease	0.74	0.001

The mean time from hospitalization to CRRT initiation was noted to be three days. With CRRT initiation on day 2 of hospitalization, a significant improvement in outcome, with a 29% decrease in mortality (N = 2,611) (p = 0.013) and a 31% reduction in hospital length of stay (p = 0.008), was noted.

## Discussion

CRRT is a choice of RRT for sepsis-associated AKI, performed in critically ill patients in ICU settings. S-AKI is at higher risk of mortality and longer hospital stays [11,12]. In our literature, we highlight that the appropriate time to initiate CRRT is between 24 and 48 hours, and we have emphasized that factors like age and HTN adversely affect the outcomes of CRRT. In contrast, patients with DM and CKD did better with CRRT.

Our study highlights that the appropriate time to initiate CRRT is between 24 and 48 hours to achieve significant improvement in mortality. There are no guidelines available on when to start CRRT in S-AKI. Gist et al. [10], Chen et al. [13], and Yoon et al. [14] suggest starting CRRT early, while Barbar et al. [15] have demonstrated no significant benefits of starting CRRT early vs. late. An et al. suggest an algorithm for when and why to start CRRT in S-AKI depending on several factors [16].

Old age is a well-known independent predictor of mortality in sepsis [17,18]. Elderly people are more prone to sepsis as they have low baseline immunity due to a lower number of T and B cells [19-21]. Additionally, delay in diagnosis is a huge con in elderly people due to the absence of typical sepsis symptoms in such age groups. Comorbidities and malnutrition also interfere with the diagnosis and therapeutic management. Different pharmacokinetics, pharmacodynamics, and polypharmacy in such individuals make it difficult to make appropriate therapeutic choices and often difficult to achieve the right combination of therapy [8]. Furthermore, family making decisions and ongoing discussions or changes in care goals may significantly impact care. Moreover, low BMI, history of sepsis, CHF, and liver cirrhosis are independent risk factors in mortality prediction [15], and the prevalence of these morbidities is higher in the elderly population.

Our study has demonstrated that the history of HTN is associated with high mortality, which was depicted in a previous study by Deng et al., as even small changes in blood pressure affect kidney dysfunction [22]. CRRT is associated with some degree of hemodynamic instability, and that significantly affects the patients who have baseline HTN and results in negative outcomes [23,24]. Hence, Yi et al. and Kim et al. suggest a high MAP goal during CRRT with S-AKI [22,25,26].

We found improved mortality outcomes in patients with a history of DM and/or CKD undergoing CRRT with S-AKI. Previously, Baccaglini et al. demonstrated a negative association between preexisting DM and sepsis mortality [27]. This finding could be supported by the evidence that suggests that DM is associated with a lower risk of acute respiratory failure and a higher risk of AKI, which, in turn, is directly managed with the CRRT, as described previously by Esper et al., Yende et al., and Moss et al. in their studies [28-30]. One other study by Venot et al. depicted that DM is not associated with AKI or the need for CRRT; however, it is associated with persistent renal dysfunction who experienced AKI during the event [31]. Another study by Zohar et al. has enlightened that the history of DM is not associated with outcomes in sepsis, but poorly controlled hyperglycemia (>200 mg/dL) is associated with poor prognosis, especially in diabetics [32].

Limitation

While the study provides important insight into predictors for outcomes in CRRT in patients with septic shock, it is not without a limitation. The NIS database captures hospitalization rather than individual patient data. NIS database captures diagnosis and comorbidities but does not capture individual patient data, clinical data points such as antibiotics, lab values, duration of CRRT, blood pressure changes, etc. We could not perform detailed analyses of the prognostic factors influencing mortality outcomes.

## Conclusions

In summary, current guidelines do not recommend any set time frame to start CRRT in S-AKI patients; we tried to highlight that when CRRT was started between 24 and 48 hours, it resulted in optimal benefit from therapy. While age and HTN were associated with high mortality with CRRT in S-AKI, DM and CKD showed opposite trends. While there is still an ongoing discussion in the literature about when and with whom to start CRRT, we sought to contribute to the ongoing trend in the national database to guide the professional community. Additionally, we recommend further dedicated trials are needed to have more standardized guidelines.
